# Eco friendly silver nanoparticles synthesis by *Brassica oleracea* and its antibacterial, anticancer and antioxidant properties

**DOI:** 10.1038/s41598-020-74371-8

**Published:** 2020-10-29

**Authors:** Sabah Ansar, Hajera Tabassum, Norah S. M. Aladwan, Mir Naiman Ali, Basmah Almaarik, Salma AlMahrouqi, Manal Abudawood, Naheed Banu, Roua Alsubki

**Affiliations:** 1 Department of Clinical Laboratory Sciences, College of Applied Medical Sciences, King Saud University, P.O. Box 10219, Riyadh, 11433 Saudi Arabia; 2 Microbiology Section, Riyadh Municipality Central Area Labs, Riyadh, Saudi Arabia; 3 Department of Nutrition and Dietetics, Sultan Qaboos University Hospital, Seeb, Oman; 4 Chair of Medical and Molecular Genetics Research, Department of Clinical Laboratory Sciences, College of Applied Medical Sciences, King Saud University, Riyadh, Saudi Arabia; 5 Department of Physical Therapy, College of Medical Rehabilitation, Qassim University, Buraydah, Saudi Arabia

**Keywords:** Biotechnology, Microbiology, Plant sciences, Medical research, Nanoscience and technology

## Abstract

Production of environmentally amenable silver nanoparticles (AgNPs) has garnered the interest of the scientific community owing to their broad application primarily in the field of optronics, sensing and extensively in pharmaceuticals as promising antioxidant, antimicrobial and anticancer agents. The current study emphases on production of ecofriendly silver nanoparticles from *Brassica oleracea* (BO) and investigated their antibacterial, anticancer and antioxidant activity. The characteristics of synthesized BO-AgNPs were studied by ultraviolet–visible spectroscopy, particle size analysis, electro kinetic/zeta potential analysis, and Transmission electron microscope (TEM). A distinctive absorption maximum at 400 nm confirmed the formation of BO-AgNPs and data on TEM analysis have shown that the synthesized nanoparticles were predominantly spherical in shape. The BO-AgNPs obtained were assessed for antibacterial, antioxidant, and cytotoxic ability in MCF-7 cells. The antibacterial activity expressed was maximum against *Staphylococcus epidermidis* (Gram positive) and Pseudomonas *aeruginosa* (Gram negative) with DIZ of 14.33 ± 0.57 and 12.0 ± 0.20 mm respectively*.* Furthermore, the ability of the synthesized green nanoparticles to scavenge free radicals revealed a strong antioxidant activity. The cytotoxicity increased proportionately with increasing concentration of the green synthesized BO-AgNPs with maximum effect at 100 μg/ml and IC50 of 55 μg/ml. In conclusion, the data obtained in the study is reflective of the role of BO-AgNPs as potential and promising antimicrobial agent against bacterial infections and potential anticancer agent in cancer therapy.

## Introduction

Nanoscience is one of the remarkable fields of science dealing with utilization and development of structures and materials with size ranging in nanometer scale. Nanoparticles (NPs) are nano-sized ranging from 1–100 nm produced through numerous methods. There are various methods for synthesis of NPs, yet these methods employ hazardous and toxic chemicals, which poses a high risk of toxicity. Other disadvantages related to these methods, is low production rate and low biodegradability^1–3^. The production of NPs employing nontoxic conditions is critical to address the increasing concerns regarding their toxicity^4^. The green chemistry utilizing plant as source for production of AgNPs is gaining importance. The usage of plants for the manufacture of NPs has gathered the interest of scientists as a quick, economical, and environmentally sustainable method^5,6^. The reduction potential of the plant is crucial in formation of nanoparticles, mediating the stabilization and reduction and their capping. Green synthesis of NPs involves three phases. In phase I, mineral ions are eluted from salts in the manifestation of plant products as reducing agents with subsequent reduction of mineral ions from bi-valent to zerovalent forms (activation phase). In the second phase (growth phase) NPs formed by the coalescence of isolated metal atoms in their reduced form undergo biological reduction. Eventually, NPs get capped by plant metabolites during the termination phase and ones with a stable morphology are procured^7–9^.

Biological synthesis of nanoparticles mediated through plants and microorganisms has been procured as excellent antimicrobials. A range of microbial species used for Ag-NP synthesis is safe, biocompatible and ecofriendly^10–14^. Green synthesis of nanoparticles from endophytic actinomycetes and Penicillium have also gained importance in nanoscience research^15^. Owing to increased antibiotic resistance to microorganisms, new antimicrobials that can reduce healthcare costs are in demand. There has been increase in requirement for these new antimicrobials at higher rates owing to its use in varying fields like medicine, environment, textiles, and cosmetics. They are primarily prepared from noble metals, e.g., silver, gold, platinum, and palladium with AgNPs being the most exploited^16^. Apart from the varied role of nanoparticles in the field of nanotechnology including the chemical kinetics, nanoelectronics, cytocompatibility studies, tissue engineering and targeted drug delivery, the applications are extended to production of potential antimicrobial and anticancer agents in the field of biomedical sciences^17–20^. Interestingly, nanotechnology with the distinctive characteristics of metal nanoparticles like high surface-to-volume ratio, ease of synthesis and surface functionalization makes prospectives in cancer therapeutics^21,22^.

*Brassica oleracea*, is a popular cruciferous vegetable belonging to the family Brassicaceae is considered a food of high nutritional value. It possess effective biological and immunological activities and is very good source of electrolytes, minerals, vitamins, dietary fibers, anti-oxidant compound (sulforaphane, flavonoids etc.) and phytochemicals like indoles which possess detoxifying property^23,24^. Nevertheless, nanoparticles synthesized from *Brassica* species have been reported earlier^25–30^, a comparative analysis of different Brassicaceae members for their reducing capacity, for controlled synthesis of AgNPs and to explore better performing varieties with higher antimicrobial potential is lacking. Hence, in the present study owing to the presence of antioxidants and other detoxifying chemicals, the NPs synthesized from *B. oleracea* has been hypothesized to play role as an effective antimicrobial and anticancer tools. Moreover, the production of AgNPs mediated from *B. oleracea* need to be evaluated for antimicrobial screening, anticancer and antioxidant properties to provide a green alternative to other chemically synthesized AgNPs. The study thus aimed to produce silver nanoparticles from *B. oleracea* leaves by green chemistry process, an alternative ecofriendly approach for its potential applications.

## Results

**Figure 1:**
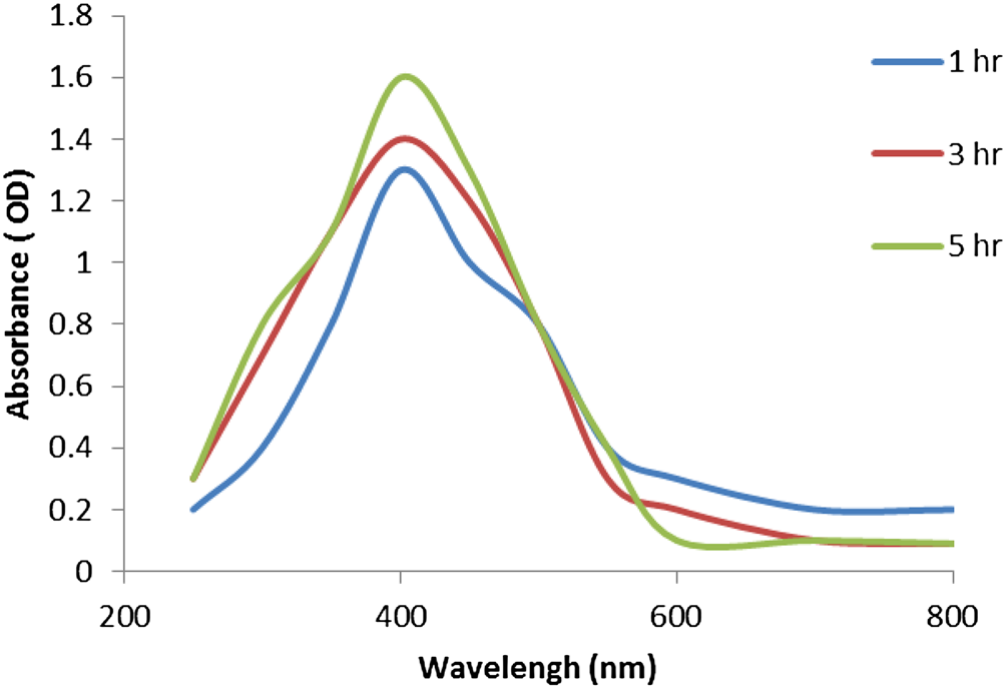
Absorption spectra of the synthesized Ag nanoparticles from *B. oleracea* leaf aqueous extract.

In this study, AgNPs production was completed by treating extract of BO leaves with silver nitrate solution. This reaction showed the color change from pale green to dark brown. A persistent λ max (at 400 nm) shown at altered time intervals reflects of the contained molecular size and configuration of the synthesized NPs (Fig. 1). This complex was responsible for changing color from greenish yellow to brown. This color change indicates the formation of Ag nanoparticles. The Ag nanoparticles synthesized in each extract solution was analyzed using UV–Vis spectroscopy. Reduction of Ag ions using BO-leaf extract is confirmed by the UV spectrum which exhibited a strong absorption peak at ~ 415 nm, which ultimately confirmed the formation of BO-AgNPs.

**Figure 2:**
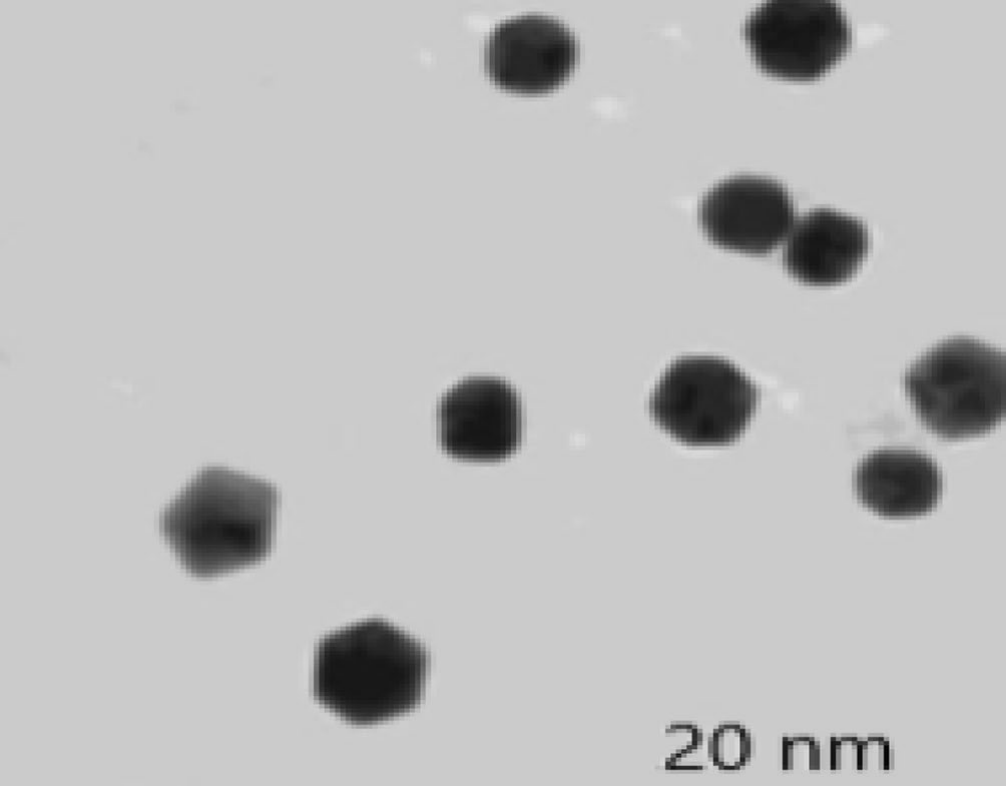
TEM image of Ag nanoparticles prepared with aqueous *B. oleracea* leaf extract.

Results of TEM image (Fig. 2) showed that the highly-densed AgNPs formed by the BO leaves further affirm the development of silver nanostructure. The images clearly show the formation of relatively spherical nanoparticles with average diameter of 20 nm, with relatively homogenous distribution.

**Figure 3:**
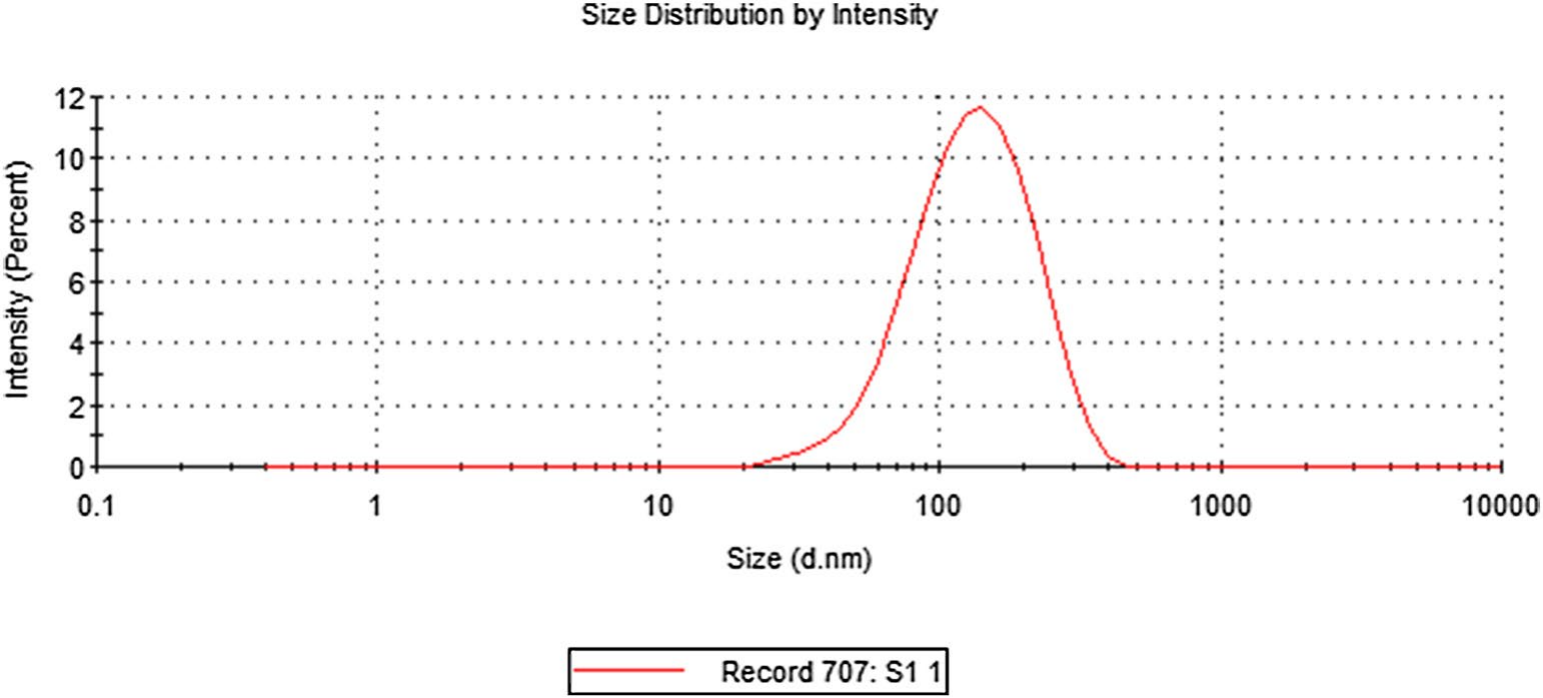
Average size of Ag nanoparticles prepared with aqueous *B. oleracea* leaf extract.

**Figure 4:**
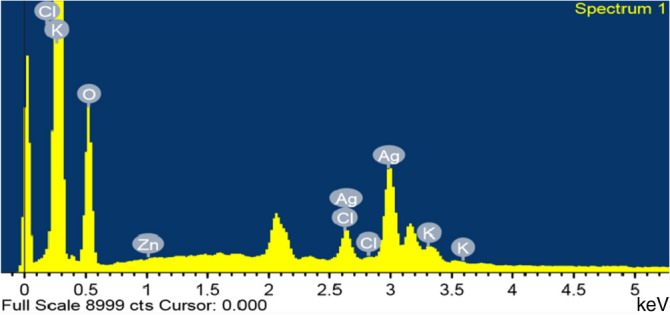
Energy dispersive X-ray elemental analysis of Ag nanoparticles prepared with aqueous *B. oleracea* leaf extract.

**Figure 5:**
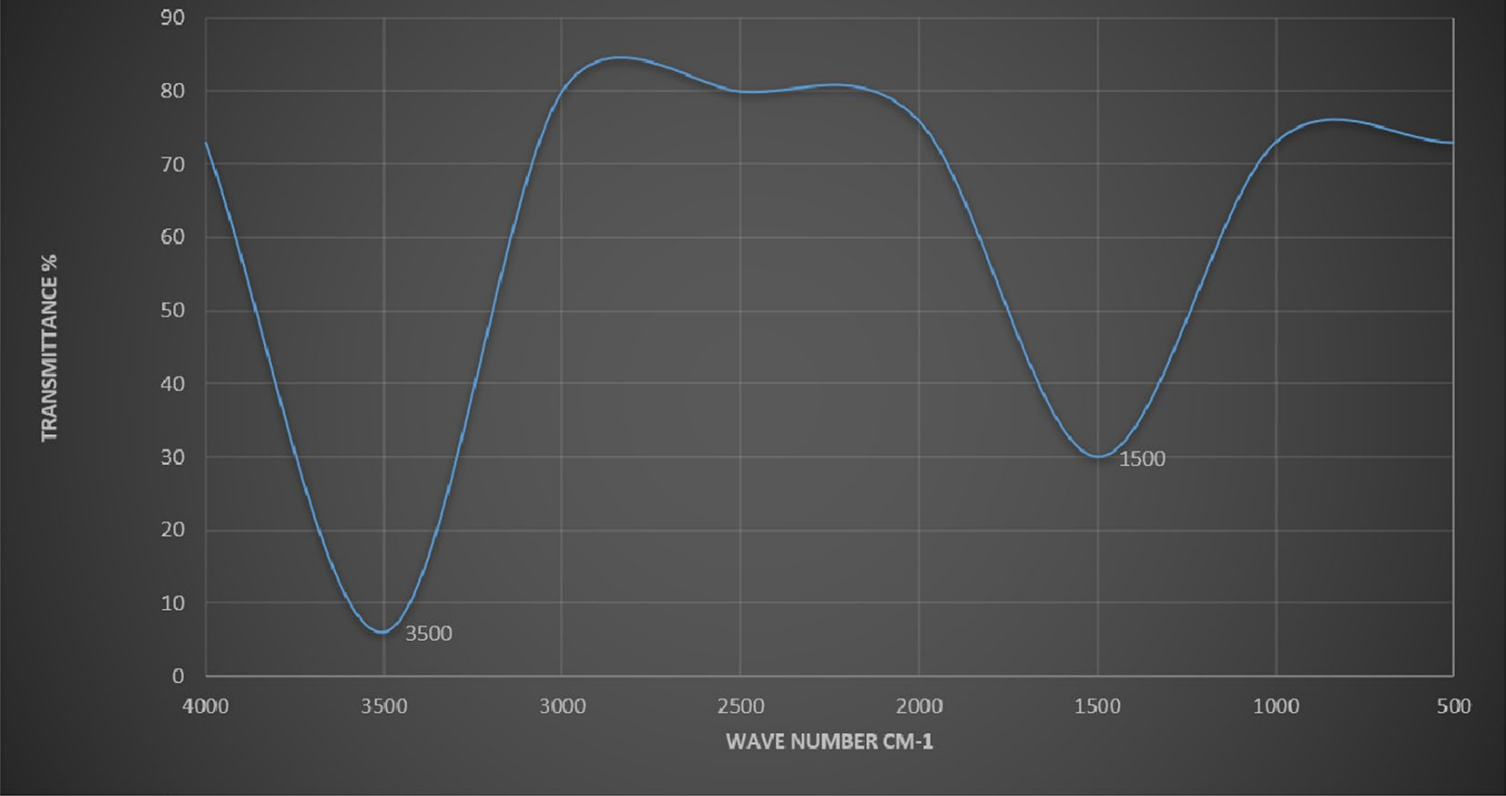
FT-IR analysis of Ag nanoparticles prepared with aqueous *B. oleracea* leaf extract.

The present study recorded a particle size of BO-AgNPs in ZS analysis as shown in Fig. 3. As a result of high sensitivity of ZS, nanoparticles showed slightly large size due to aggregation. Figure 4 depicts the EDX spectrum, revealing the clear elemental composition profile of the green synthesized BO-AgNPs. The EDX profile demonstrated a remarkable defined silver peak along with distinguishable prominent oxygen, chloride, and potassium signals. Notably, the intense signal at 3 keV strongly confirms that the major element was silver. The other signals in the range of 0.0–0.5 keV represent the typical absorption of oxygen, chlorine and potassium and thus indicates the presence of the plant extract (as a capping ligand) on the surfaces of the NPs that tends to bound AgNPs surface during extraction. Figure 5 depicts the FTIR profile, with strong absorption peaks for O–H groups of phenols and C–H aromatic stretch of groups at ~ 3500 cm^−1^ and 1600 cm^−1^ respectively.

**Figure 6:**
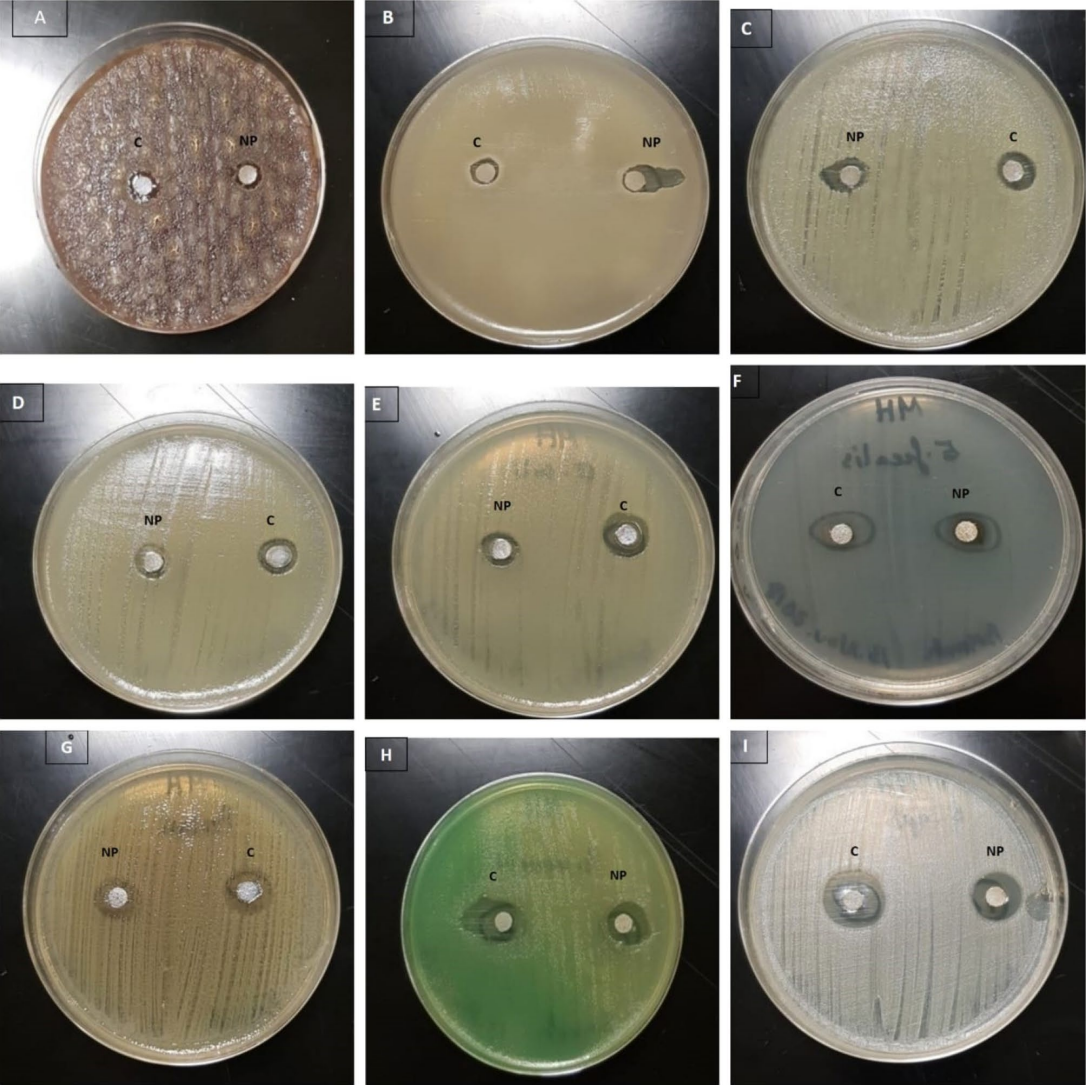
Antimicrobial activity of Ag nanoparticles prepared from *B. oleracea* against (**A**) *Bacteroides fragilis* ATCC 25285; (**B**) *Streptococcus pneumonia* ATCC 10015; (**C**) *Staphylococcus aureus* ATCC 6538; (**D**) *Klebsiella pneumonia* ATCC 10031; (**E**) *Escherichia coli* ATCC 25922*;* (**F**) *Enterococcus faecalis* ATCC 33186; (**G**) *Proteus mirabilis* ATCC 12453; (**H**) *Pseudomonas aeruginosa* ATCC 9027; (**I**) *Staphylococcus epidermidis* ATCC 12228.

The synthesized BO-AgNPs were evaluated for antibacterial activities towards Gram-positive and Gram-negative bacterial strains by the inhibition zones and MIC. About nine varied strains of bacteria were used for the antibacterial efficiency of the synthesized BO-AgNPs as shown in Fig. 6. Results of disc diffusion assay expressed as diameter of inhibition zone (DIZ) (Table 1). Minimum inhibitory concentration was also determined for synthesized nanoparticles and the results are shown in Table 2.

**Table 1:** Antibacterial activity of biosynthesized silver nanoparticles using disc diffusion assay.

Bacterial strains	Zone of inhibition (mm)	
	BO-AgNP	Control
*Bacteroides fragilis*	9.43 ± 0.40	9.56 ± 0.30
*Streptococcus pneumoniae*	10.33 ± 0.57	11.00 ± 0.20
*Staphylococcus aureus*	10.1 ± 0.17	10.78 ± 0.11
*Klebsiella pneumoniae*	10.0 ± 0.50	11.00 ± 0.20
*Escherichia coli*	10.0 ± 1.00	10.8 ± 0.50
*Enterococcus faecalis*	11.16 ± 0.28	11.45 ± 0.15
*Proteus mirabilis*	11.33 ± 0.57	12.00 ± 0.25
*Pseudomonas aeruginosa*	12.0 ± 0.20	12.5 ± 0.15
*Staphylococcus epidermidis*	14.33 ± 0.57	14.59 ± 0.67

**Table 2:** Minimum inhibitory concentration of AgNPs synthesized by *Brassica oleracea* on tested bacterial strains.

Bacterial strains	Minimum inhibitory concentration (µg/ml)	
	BO-AgNPs	Positive control
*Bacteroides fragilis* ATCC 25285	50	25
*Streptococcus pneumoniae* ATCC 10015	25	25
*Staphylococcus aureus* ATCC 6538	25	12.5
*Klebsiella pneumoniae* ATCC 10031	25	12.5
*Escherichia coli* ATCC 25922	25	12.5
*Enterococcus faecalis* ATCC 33186	12.5	6.25
*Proteus mirabilis* ATCC 12453	12.5	6.25
*Pseudomonas aeruginosa* ATCC 9027	12.5	3.1
*Staphylococcus epidermidis* ATCC 12228	6.25	6.25

**Figure 7:**
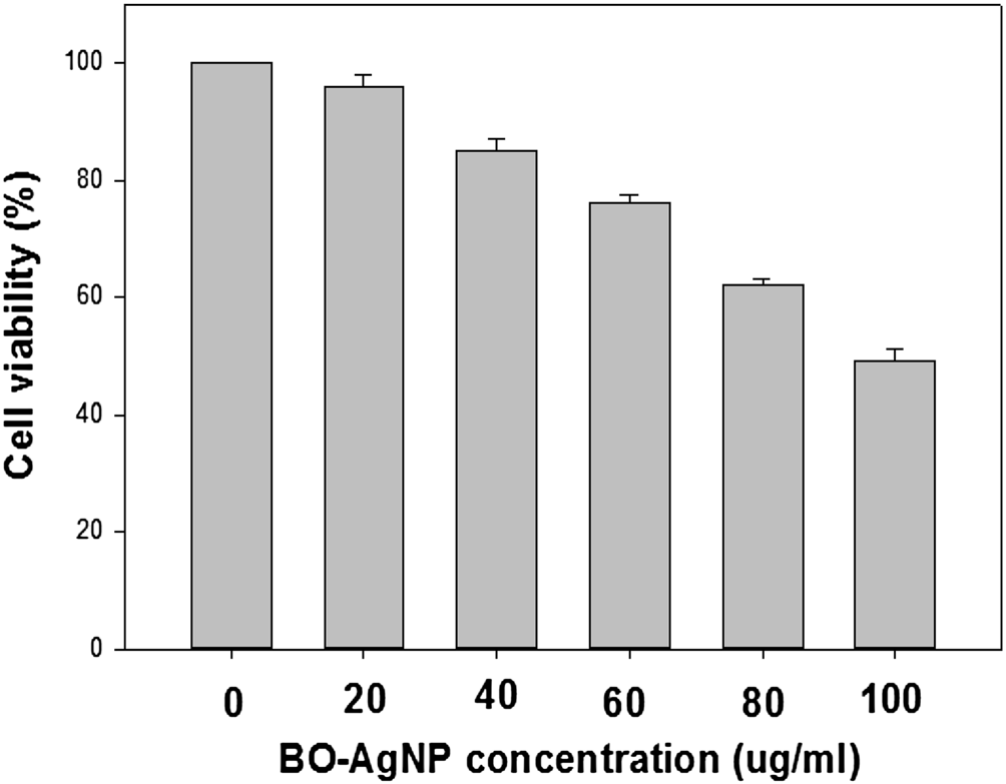
Effect of BO-AgNPs on cell viability of MCF-7 cell lines by MTT assay.

The cytotoxicity of synthesized AgNPs was observed following reduction of 3-(4,5-dimethylthiazol-2-yl)-2,5-diphenyl tetrazolium bromide dye (MTT). Post 24-h treatment of MCF-7 cells with BO-AgNPs, revealed that the viability of cells is dependent on the dosage of biosynthesized AgNPs thus reflecting the antagonistic effect upon the cancer cell line. MCF-7 cells treated with *B. oleracea* capped silver nanoparticles, exhibited cell death at higher concentrations as shown in Fig. 7. Each result represents the mean viability ± standard deviation (SD). Cell viability was calculated as the percentage of viable cells compared to untreated controls. It was observed that the cytotoxicity increased proportionately with increasing concentration of the green synthesized BO-AgNPs with maximum effect at 100 μg/ml with IC-50 of 55 μg/ml.

### Antioxidant activity of BO-AgNPs

**Figure 8:**
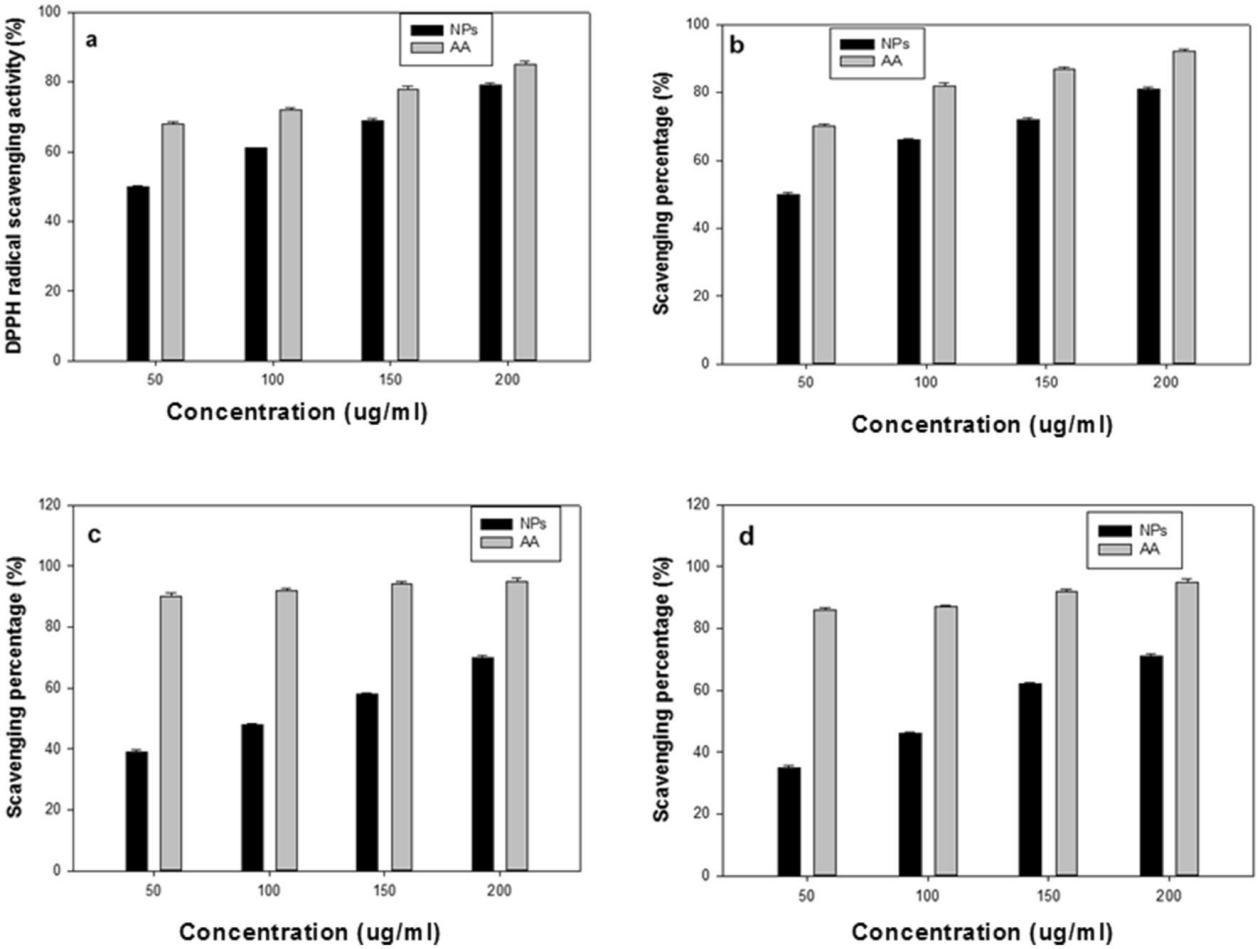
Free radical scavenging ability of BO-AgNP_S_: (**a**) DPPH assay; (**b**) nitric oxide–scavenging activity; (**c**) superoxide activity; (**d**) hydroxyl-scavenging activity of BO-AgNPs with respective standards. *AA* ascorbic acid.

The antioxidant activity of the green synthesized BO-AgNPs was evaluated using DPPH, nitric oxide, superoxide and hydroxyl radical and are depicted in Fig. 8a–d. The DPPH scavenging activity of BO-AgNPs using ascorbic acid as standard antioxidant is shown in Fig. 8a. As reflected from the figure, the antioxidant properties of the samples increased with increasing concentrations of BO-AgNPs in the range 50–200 μg/ml resulting in increase in percentage DPPH radical scavenging abilities. A dose dependent pattern was observed in the scavenging activity, which increased with increase in dose of the BO-AgNPs. Nevertheless, the scavenging potential of the BO-AgNPs was comparatively lower than that of the standard. A concentration of 200 μg/ml for BO-AgNPs exhibited the highest antioxidant activity (79%). Furthermore, the IC 50 value of BO-AgNPs around 50.37 μg/ml comparable with IC50 value of ascorbic acid 44.10 μg/ml demonstrated the potential radical scavenger properties of BO-AgNPs. Also, antioxidant activity by nitric oxide assay was found effective in range of 50–81% for the above-mentioned concentrations (Fig. 8b).The superoxide radical and hydroxyl scavenging activity of BO-AgNPs were recorded around 70% and 35–71% respectively at a higher concentration of 200 μg/ml as depicted in Fig. 8c,d. As evident from the above scavenging experiments, the synthesized nanoparticles demonstrated to possess promising antioxidant properties.

## Discussion

The synthesis of AgNPs has garnered the interest of the scientific community owing to their numerous applications in optronics, sensing, catalysis and pharmaceuticals. The green synthesis of NPs using plant extracts has more advantages than using microorganisms because it is a single-step method, is nonpathogenic and economic. Several plants and weeds have materialized as probable sources reducing silver nitrate in the synthesis of AgNPs^31–33^. The composition of organic metabolites differs among plants growing under varied climatic conditions. Consequently, there exists distinct variation in the characteristics of biologically produced AgNPs^34–36^.

Owing to the phenomenon of Surface Plasma Resonances (SPR), metal nanoparticles including silver exhibit absorption in the UV–Visible region of the electromagnetic spectrum^37^ therefore helpful in monitoring the Ag+ reduction. A strong absorption at ~ 415 nm is reflective of reduction of Ag+ ions by the BO leaf extract and ultimately the production of BO-AgNPs. Interestingly, the peak/absorption maxima provides information on the morphology of the synthesized nanoparticles. Absorption maxima at lower wavelength is indicative of smaller size NPs and broader peak/higher wavelength is typical of enlarged particle size. The absorption peak at relatively lower wavelength (415 nm) obtained in the present study points towards the formation of smaller size nanoparticles. AgNPs prepared with aqueous BO leaf extract show higher sensitivity of ZS showing fewer amounts of large particles and increased size of the ZS-measured particles due to aggregation. The findings are in agreement with previous studies in which a similar difference was reported^38^. EDX elemental analysis substantiates the occurrence of AgNPs in pellets obtained and the existence of other elements adhering on the AgNPs reflected the plant based origin of these biomolecules. The crystalline nature of AgNPs was clearly seen from the EDX pattern formed by the reduction of BO-AgNPs. A strong signal peak for silver was observed at 3 keV, confirms the formation of AgNPs. This absorption peak approximately at 3 keV is due to surface plasmon resonance exhibited by silver^39^. The current observation is in line with the previous reports of Shankar et al. (2004) where a thin layer of organic material encircling the green-synthesized NPs was reported^40^. Furthermore, FTIR measurements were carried out in order to identify the presence of various functional groups in biomolecules responsible for the bioreduction of Ag and capping/stabilization of silver nanoparticles. Figure 3 shows the FTIR profile, with strong absorption peaks for O–H groups of phenols and C–H aromatic stretch of groups at ~ 3500 cm^−1^ and 1600 cm^−1^ respectively.

AgNPs from *B. oleracea* as bactericidal agents was also investigated in the current study and results of antimicrobial activity is shown in Fig. 6. Based on the inhibition zone obtained, it was observed that the synthesized AgNPs expressed antibacterial activity in the range of 9–14 mm against *Bacteroides fragilis and Staphylococcus epidermidis* respectively*.* Highest antibacterial activity was seen against *Staphylococcus epidermidis*, which was the most sensitive to the AgNPs with a DIZ value of 14.33 ± 0.57 mm, followed by *Enterococcus faecalis* and *Proteus mirabilis* with DIZ value of 11.16 ± 0.28 mm and 11.33 ± 0.57 mm respectively. With respect to Gram negative bacteria, highest antibacterial activity was expressed against *Pseudomonas aeruginosa* (12.0 ± 0.20 mm), followed by *Escherichia coli* (10.0 ± 1.0 mm), *Klebsiella pneumoniae* (10.0 ± 0.50 mm) and *Bacteroides fragilis* (9.43 ± 0.40 mm). The observed antibacterial activity of synthesized BO-AgNPs in the current study is in accordance with previous studies^14,15^. Anandalakshmi et al. (2016) observed silver nanoparticles mediating similar bactericidal action using *Pedalium murex* leaf extract^41^. Green synthesis was found to be eco-friendly and biosynthesized AgNPs possessed antibacterial activity against *E. coli, Klebsiella pneumoniae, Mariniluteicoccus flavus, Pseudomonas aeruginosa, Bacillus subtilis, Bacillus. pumilus* and *S. aureu*s. Supportingly, Tamileswari (2015) observed antibacterial activity of AgNPs against pathogenic bacterial strains like *K. pneumonia, B. subtilis, S. aureus *and* E. coli*^23^. The results obtained for Gram negative strains with maximum antibacterial activity against pseudomonas are in line with previous study by Kumar et al. (2012)^42^.

Furthermore, results obtained from Minimum inhibitory concentration (MIC) of synthesized BO-AgNPs are in accordance with antibacterial activity by agar diffusion method. The lowest MIC of 6.25 µg/ml was recorded for *Staphylococcus epidermidis* ATCC 12228, against which highest zone of inhibition (14.33 ± 0.57) was observed (Table 2). Similarly, highest MIC of 50 µg/ml was observed against *Bacteroides fragilis* ATCC 25285, with lowest zone of inhibition recorded (9.43 ± 0.40). On contrary, MIC values recorded for positive control were at lower concentration, as pure AgNO3 was used. Slight variation was observed with lower MIC values recorded for *Pseudomonas aeruginosa* ATCC 9027 (3.1 µg/ml) and highest values recorded for *Bacteroides fragilis* ATCC 25285 and *Streptococcus pneumoniae* ATCC 10015 (25 µg/ml). The results for MIC indicate that the synthesized BO-AgNPs are effective at a low concentration to inhibit the bacterial growth.

The growth inhibition by the AgNPs is primarily due to the formation of pores in the bacterial cell wall accompanied with changes in cell membrane permeability due to deposition of AgNPs at these sites. Several underlying mechanisms involved in antibacterial action of AgNPs have been proposed. Possibly, the disruption of the thiol group in the electron transport chain enzymes followed by the binding of the AgNPs to the cell wall and membrane of bacterial cell accounts for the inhibitory effect of these silver nanoparticles. The metallic NPs interact with bacterial cell wall through attraction between the microbial cell wall’s negative charge and NPs’ positive charge. Due to this interaction, the permeability function of the cell membrane changes and, hence, the bacterial integrity disrupts and causes cell death^43^. Additionally, the increased affinity of silver nanoparticles to elements like sulphur and phosphorus owe to the antibacterial property of these NPS. These elements are abundantly found in the bacterial cell wall and membranes. Silver nanoparticles are known to inhibit the absorption of phosphorus and altering the levels of phosphate, mannitol in the bacterial cell^44^. The viability of bacterial cell is reduced due to interaction of AgNPs with proteins containing sulfur in or outside of the cell membrane^45^. The broad-spectrum antibacterial activity of these AgNPs is due to combinatorial action^46^. A three way antibacterial mechanisms involves: (a) the uptake of silver ions by bacteria leading to interruption of ATP production and DNA replication, (b) AgNPs can trigger the production of free radicals generating oxidative stress, (c) AgNPs can directly damage the bacterial cell membranes leading to lysis of the cell^47^. The colonization of bacterial species like *S. epidermidis* and *E. faecalis* in central venous catheters poses a greater risk of catheter associated blood infections. The green-synthesized AgNPs could be manifested to minimize such catheter-associated infection, which poses higher cost for heathcare^48–52^. The prevalence of *S. pneumoniae* infections in ICUs can thus be minimized by adopting such sterilization procedures^53^. Furthermore, owing to its antimicrobial property, synthesized BO-AgNPs can also be used in food packaging as nanosensors to detect the microbial contamination.

AgNPs also plays a key role in tumor regulation via their cytotoxic effects. Cytotoxicity of AgNPs is mediated by the action of Ag+ ions. AgNPs facilitate the formation of reactive oxygen species (ROS), superoxide synthesis following reduction of oxygen by electron from electron transport chain on the mitochondrial surface. The generated reactive oxygen species thus leads to oxidative damage of cellular contents including DNA, proteins and lipids and consequently results to cell death^54,55^. The cytotoxicity increased proportionately with increasing concentration of the green synthesized BO-AgNPs with maximum effect at 100 μg/ml suggesting their use as an alternative chemotherapeutic agent. The anticancer potential exhibited by the BO-AgNPs in the present study are in accordance with previous evidences on MCF 7 cell lines, Hep-2 cells and Hela cell lines from biosynthesized AgNPs from leaf extract of *Annona squamosa, Piper longum* and *Morinda citrifolia* respectively^56–58^. The anticancer property of BO-AgNPs demonstrated by cytotoxic assay could be due to presence of compounds like sulforaphane and indole-3-carbinal, which boosts DNA repair in cells and appears to block the growth of cancer cells. Moreover, the phytonutrients and antioxidants play a chemo-protective role against oxidative stress-related diseases such as cancer.

Human body is under vulnerable attack by the free radicals causing cellular damage, which is generally being neutralized by the antioxidant molecules. Majority of antioxidants are known to decelerate the progression of chronic diseases. Cellular oxidation which is crucial for the growth of the cell is associated with some adverse effects due to the generation of free radicles and reactive oxygen species. These free radicals in surplus amounts lead to devastating effect on the protective antioxidant enzymes like superoxide dismutase, catalase and peroxidase resulting in cellular damage by oxidizing vital biomolecules, subsequently leading to apoptosis/cell death^59^. The antioxidant efficiency of BO-AgNPs tested against various free radicals yielded intriguing results in this study. Presence of phytochemicals like flavonoids with several hydroxyl groups and phenolic functional groups on the surface as capping agents on these nanoparticles may account for the observed antioxidant capacity. The observed antibacterial, antioxidant and cytotoxic activity of the silver nanoparticles evidenced in the present study are in accordance with previous studies^60,61^.

## Conclusion

The synthesized silver nanoparticles from *Brassica oleracea* (BO) by green chemistry demonstrated an enhanced antibacterial, anticancer, and antioxidant activity. The applicability of nanoparticles is extendable to various fields like environment, biomedical, and electrochemistry due to lack of toxic reagents in the process of green synthesis. In conclusion, synthesis of Ag-NPs by green process has emerged as promising tools in extension of the plant mediated nanocarriers in the field of tumor diagnosis, drug delivery systems and radiological imaging.

## Methodology

### Processing of BO leaves

Fresh green leaves of *Brassica oleracea* (BO) had been washed with double-distilled water and subsequently air-dried at 20 °C. Later these leaves (around twenty grams) were boiled in 250 ml of ultrapure water for 20 min to prepare the leaf extract, and cooled. The prepared BO-AgNPs were stored until further investigation at 4 °C.

### Preparation of silver nanoparticles (green synthesis)

Silver nitrate (AgNO_3_) from Sigma-Aldrich with ≥ 99.5% purity was utilized. 5 ml of the leaf extract was collected for reduction of 1 mM Ag-NO_3_ (50 ml) aqueous solution at ambient temperature for 10 min, resulting in a brownish yellow solution, thus demonstrating the development of AgNPs. UV–Vis Spectrophotometer was used to monitor the synthesized nanoparticles for 5 h.

### Characterization of synthesized nanoparticles

The following protocols have been used to characterize the synthesized leaf extract AgNPs.

#### Detection of BO-AgNPs by UV–Vis spectrophotometry

UV–Vis spectrophotometer (LIUV-310, Lambda Scientific, Australia) was employed to measure the Surface Plasmon Resonance (SPR), resulting due to production of silver nanoparticles typically from direct reduction of silver nitrate by BO extract following method of Shanker et al. (2005)^59^. The analysis was performed in quartz cuvettes using distilled water as a reference solvent. Ag-NP samples for UV–Vis analysis were diluted in 1:4 ratio; prepared by diluting 2 ml of Ag-NP solution in 8 ml of distilled water. Absorption spectra was obtained by sampling approximately one milliliter of the suspension at different time intervals (1, 3, and 5 h) to track the completion of bioreduction of Ag+ ions.

#### Particle size and zeta potential (ZP) analysis

The Zetasizer (ZS) Nano Series ZS (model number ZEN3600) determined the hydrodynamic size and zeta potential of the AgNPs following method of Erdogan et al. (2019)^62^. The red laser (wavelength-633 nm) is incidented at an scattering angle of 173° in a medium with viscosity of 0.887 milli Pascals at 25 °C to analyze the size of the particle. The zeta potential value facilitates the understanding of interparticle forces of interaction. The ZP value affects the constancy of colloidal system. Low-density, small particles in suspension with high + /− zeta potentials deter each other.

#### Energy dispersive X-ray (EDX) spectroscopic analysis

JEOL JEM 2100 high-resolution was used for EDX analysis of BO-AgNPs. The presence of elemental silver was validated through EDX with detection of other elements in the particle. Energy dispersive analysis X-ray spectrometer works utilizing the properties of photon; unit of light. The energy of single photon in the X-ray region produces a measurable pulse X-ray. X-ray, besides processing electronics are detected by semiconductor material for spectral analysis. The EDX observations were recorded coupled with SEM^62^.

#### Evaluation of antibacterial activity

The bactericidal property of the synthesized BO-AgNPs were investigated on selected bacterial strains procured from American Type Culture Collection Center (ATCC). Both Gram-positive and Gram-negative strains including Bacteroides fragilis ATCC 25285, Pseudomonas aeruginosa ATCC 9027, Staphylococcus aureus ATCC 6538, Enterococcus faecalis ATCC 33186, Streptococcus pneumoniae ATCC 10015, Proteus mirabilis ATCC 12453, Klebsiella pneumoniae ATCC 10031, Escherichia coli ATCC 25922, and Staphylococcus epidermidis ATCC 12228 were tested. Inoculum was prepared as per CLSI M02-A12^63^. For preparation of inoculum, isolated colonies of each bacterial culture were selected from 18–24 h incubated agar plates and inoculated in tryptone soya broth (Oxoid, UK) to make a suspension. The turbidity of the suspension was adjusted to achieve a CFU of 1.0–2.0 × 10^8^ CFU/ml (CLSI) by UV–Visible Spectrophotometer (UV 1800, Shimatzu, Switzerland) at 600 nm. 0.1 ml of each bacterial culture suspension was inoculated on Mueller Hinton agar (Oxoid, UK) plates and evenly spread with a sterile spreader. Sterile paper disc of 6 mm diameter containing 25 μg/ml silver nanoparticles along with AgNO_3_ (30 μg/ml) containing discs was placed in each plate as control. Antibacterial activity was tested by standard Kirby–Bauer Method^64^, Mueller Hinton agar was employed and Petri dishes (90 mm) containing 18 ml of Mueller Hinton agar were seeded with 100 μl inoculum (approximately 10^8^ CFU/ml) of each bacterial strain in Mueller Hinton broth.

The inoculum of each bacterial culture were evenly spread on petri dishes and discs previously saturated were placed on petri dishes with culture. All petri dishes were kept at 37 °C for 18–24 h for incubation and diameter of zone of inhibition (DIZ) was calculated. The experiment was run in triplicate and DIZ value were reported as mean ± SD values.

#### Determination of minimum inhibitory concentration (MIC)

MIC of the synthesized NPs was determined by Macrodilution method in sterile test tubes as per CLSI 07-08^65^. Serial dilutions of synthesized NPs were made from a concentration of 1 mg/ml to get a concentration of 100 µg/ml to 50, 25, 12.5, 6.25, 3.1, 1.5 and 0.78 µg/ml. Standard AgNO3 was also tested for determination of MIC in the same range of concentration for comparative analysis. The inoculum for test strains containing 5 × 10^5^ CFU/ml was prepared in a similar manner as for antibacterial activity testing.

#### Cytotoxicity assay

Eagle minimum essential medium (EMEM) containing 10% FBS was used to culture Human breast cancer cell lines (MCF-7). The following conditions were maintained in incubator- 95% air, 100% relative humidity, 5% CO_2_ and temperature 37 °C. To obtain a final density of 1 × 10^5^ cells/ml, viable cells were enumerated using a hemocytometer, with 5% FBS as diluents. 96 well polystyrene-coated plate (flat bottomed) was overlaid with MCF-7 cells (in growth phase) and incubated for cell attachment. AgNPs at varying concentrations: 0, 20, 40, 60, 80, 100 µgm/ml was added to the seeded cell lines and treated for 48 h. Phosphate buffered saline (PBS) containing MTT (3-(4,5-Dimethylthiazol-2-yl)-2,5-diphenyltetrazolium bromide 5 mg/ml) reagent was added after 24 h and incubated further for 3 h at 37 °C. The medium served as control. Post incubation absorbance was read in micro plate reader at 570 nm. The cell viability percentage was then determined with respect to control^66^.

#### Evaluation of antioxidant assay

##### DPPH assay

The antioxidant property of synthesized BO-AgNPs was determined by DPPH-radical scavenging assay. BO-AgNPs prepared at varying concentration—50, 100, 150 and 200 μg/ml were thoroughly agitated and mixed with 0.1 mM DPPH. The solution was incubated in dark for 15 min. The decrease in the concentrations of DPPH was monitored by measuring absorbance at 517 nm^67^. Later, the antioxidant capacity of BO-AgNPs calculated using ascorbic acid as the standard. The DPPH scavenging ability was expressed in % as follows.

$$ {\text{DPPH }}\;{\text{scavenging}}\;{\text{ assay}}\;{\text{ percentage }} = {\text{ Control}}\;{\text{ absorbance }}{-} \, ({\text{Sample}}\;{\text{absorbance }}{-}{\text{blank }}\;{\text{absorbance}}) \, /{\text{control }}\;{\text{absorbance }} \times \, 100 $$

##### Nitric oxide radical-scavenging assay

For determination of nitric oxide scavenging activity, BO-AgNPs at different concentrations were mixed with 2 ml Fe-NPs and incubated at room temperature for 150 min. Following incubation, around 0.5 ml aliquot of the incubated mixture was mixed with 1 ml of sulfanilic acid (0.33% sulfanilamide in 20% acetic acid) and incubated further for 5 min. Subsequently, 0.1% naphthyl ethylenediamine dihydrochloride was added to the above reaction solution and incubated for additional 30 min at the ambient temperature and the absorbance read at 546 nm^68^. The scavenging percentage was calculated as follows:

$$ \% \;{\text{scavenging/reduction }} = \, ({\text{T}}_{0} - {\text{T}}) \, = {\text{T}}_{0} \times 100; $$

where T_0_—absorbance of the control, T—absorbance of the test sample.

##### Superoxide anion radical-scavenging assay

Concisely, about 1 ml reaction mixture containing 100 mM phosphate buffer (pH 7.4), 468 μM NADH, 156 μM NBT and 60 μM PMS was prepared and mixed with varying concentrations of nanoparticles ranging from 50–200 μg. The reaction mixture was further incubated at room temperature for 5 min. Superoxide anion radical-scavenging assay was determined by measuring the formation of the purple formazan (nitroblue tetrazolium) detectable at 560 nm in spectrophotometer. The product is formed after detoxification of superoxide radicals generated from NAD (nicotinamide adenine dinucleotide) by BO-AgNPs^69^.

##### Hydroxyl radical-scavenging assay

Briefly, a reaction mixture volume of about 3 ml was prepared containing 1 ml each of 9 mM salicylic acid, 9 mM ferrous sulfate and hydrogen peroxide and mixed following addition of 1 ml of synthesized BO-AgNPs. After incubation, for 60 min at 37 °C in boiling water bath the absorbance of the resulting solution was measured at 510 nm. Experiment was repeated running negative control and the percentage of hydroxyl radical scavenging activity for test samples was calculated^70^.

### Statistical analysis

Statistical analysis was performed with one-way analysis of variance (ANOVA). For statistical analyses the SPSS 17.0 software was used. Three replications for each of the experiments and assays were conducted (n = 3). A mean of the three values reported in each case.
